# Preventing Childhood Sexual Abuse Related Mental Health Deterioration Using a Narrative Family Intervention in Burundi

**DOI:** 10.1007/s10802-025-01328-8

**Published:** 2025-05-10

**Authors:** Julia Schneider, Anja C. Rukundo-Zeller, Manassé Bambonyé, Jean-Arnaud Muhoza, Thierry Ndayikengurukiye, Lydia Nitanga, Amini Ahmed Rushoza, Anselm Crombach

**Affiliations:** 1 Department of Psychology, Clinical Psychology and Psychotherapy for Children and Adolescents, Saarland University, P.O.Box 15 11 50, 66041 Saarbrücken, Germany; 2 Psychology, Clinical Psychology and Clinical Neuropsychology, University of Konstanz, Konstanz, Germany; 3 Department of Clinical Psychology, Université Lumière de Bujumbura, B.P.: 1368, Bujumbura, Burundi; 4 Non-Governmental Organization Psychologues Sans Frontières Burundi, Bujumbura, Burundi; 5 Non-Governmental Organization Vivo International E.V, P.O.Box 5108, 78457 Konstanz, Germany

**Keywords:** Childhood sexual abuse, Preventive narrative family intervention, Mental health, PTSD, Parental acceptance

## Abstract

Despite the severe impact of childhood sexual abuse (CSA) on trauma-related disorders, preventive interventions are scarce, especially in (post-)conflict regions. We developed and evaluated a narrative family communication approach for sexually abused Burundian female children and adolescents (*N* = 102). The intervention cohort (*n* = 55) received psychoeducation, parental skill training, and preventive narrative exposure therapy. Intervention participants reported improved parental acceptance at 3- and 12-month follow-ups (3mFUP, 12mFUP), and showed greater improvement in overall mental health between initial assessment (IA) and 3mFUP (*d*_*rm*_ = -0.70) and between IA and 12mFUP (*d*_*rm*_ = -1.36) compared to non-treated controls (*d*_*rm*_ = -0.33, *d*_*rm*_ = -0.02, respectively). Moreover, PTSD symptoms were significantly less pronounced in the intervention cohort than in the control cohort at both follow-ups. Our findings suggest that the preventive narrative family intervention might effectively buffer against devastating mental health consequences, including emerging PTSD symptoms, in the aftermath of CSA. The study and its outcome measures were preregistered at Clinical Trials (https://clinicaltrials.gov/) with the registration number NCT05136105.

Childhood sexual abuse (CSA) is a global occurrence that is particularly prominent in (post-) war and conflict regions (Arieff, 2011). Research within the past twenty years has established a substantial association between CSA and severe deteriorations in mental health and social functioning in children and adults (Batchelder et al., 2021; Collin-Vézina et al., 2013; Cutajar et al., 2010; Schalinski et al., 2016). According to estimated population attributable risk calculations, CSA accounts for about 13% of all adult psychopathologies (Cutajar et al., 2010; Fergusson et al., 2008). One of the most common mental health disorders in the aftermath of CSA is posttraumatic stress disorder (PTSD). In a sample of 4,208 females, abused individuals were 7.25 times more likely to meet lifetime diagnostic criteria for PTSD than those who were not abused (Cutajar et al., 2010).

Considering the severe consequences of CSA and the limited access to mental health care in low- and middle-income countries, there is a great need for short but effective interventions preventing the deterioration of mental health in survivors. However, classical debriefing within 3 days following traumatic incidents yielded mixed, and partially harmful results regarding the prevention of PTSD in patients aged 16 and older (Rose et al., 2002, 2003). Yet, early interventions based on trauma-focused psychotherapeutic treatment approaches showed promising results in adult survivors of sexual violence, even though methodological limitations prevent definite conclusions about the safety of early interventions after sexual assault (Dworkin & Schumacher, 2018; Oosterbaan et al., 2019). Following the suggestion of Oosterbaan et al. (2019) “not to shy away from this field but instead invest in the exploration and further development of effective interventions to prevent PTSD in victims of sexual assault “ (p.10), we sought to develop a preventive approach for child survivors of sexual assault in low- and middle-income countries. In line with other promising preventive interventions, we grounded our approach in the theoretical and practical aspects of an existing trauma-focused psychotherapy, which has proven effective in low- and middle-income countries and (post-)conflict regions: Narrative Exposure Therapy (NET; Schauer et al., 2011; Siehl et al., 2021). More specifically, we integrated elements from a preventive version of NET (PreNET; Crombach et al., 2015), in a family communication approach. PreNET has been previously developed and implemented with Burundian soldiers. It aims at processing a singular traumatic event whilst fostering understanding of its’ autobiographic context within a person’s life. In summary, we focused on three main factors that contribute to the development and maintenance of PTSD: (1) the fear network and a possible building block effect, (2) avoidant behaviors by the child and the family, (3) parental invalidation in the aftermath of sexual violence.

According to neurobiological evidence, the development of PTSD is associated with altered memory consolidation during and after CSA. Acute stress reactions during traumatic events cause the detachment of sensory-emotional information from its original context (Brewin et al., 2010; Elbert & Schauer, 2002). Consequently, event-related sensory-emotional information are stored separately from their contextual information, forming a mutually excitatory associative network known as the fear network (Elbert & Schauer, 2002). As CSA rarely occurs as a single isolated event (Coker et al., 2000) and traumatic experiences share sensory-emotional information, the fear network expands through accumulated exposure to additional traumatic experiences (Elbert & Schauer, 2002; Schauer et al., 2011). Through mutually excitatory connections, the activation of one item within this network triggers the recall of all associated memories. Due to the lack of contextual information, this activation manifests as typical symptoms of posttraumatic stress, such as intrusions and hypervigilance (Elbert & Schauer, 2002). NET aims to disrupt the fear network by narrating the traumatic event in detail and recalling event-related emotions together with the contextual information, thereby reintegrating the two memory systems (Schauer et al., 2011). Dissolving the fear network in the aftermath of sexual assault might prevent further PTSD symptoms.

Negative cognitive appraisals of the traumatic event and its consequences contribute to acute feelings of danger. Trying to prevent feelings of acute threat, survivors tend to engage in avoidant coping strategies (Ehlers & Clark, 2000). While avoidance seems to reduce recurrent feelings of acute threat in the short term, it hampers thorough processing and reappraisal of the traumatic event in the long run (Dunmore et al., 2001; Ehlers & Clark, 2000; Meiser-Stedman, 2002). As a result, avoidance contributes to the perseverance of PTSD symptoms (Batchelder et al., 2021; Nandi et al., 2020; Ullman & Peter-Hagene, 2014). Indeed, disclosing and talking about CSA proves difficult for many children and adolescents due to abuse-related feelings of shame, guilt, and fear for self or others (Winters et al., 2020). Furthermore, children and adolescents might downplay their suffering to avoid upsetting their caregivers. The latter might avoid the subject because they feel guilty and ashamed for not being able to protect their child (Gopalan et al., 2010). Ultimately, these strategies are likely to preclude the ability of caregivers to effectively support their children and might reinforce negative stereotypes rooted in the belief in a just world, leading to victim blaming towards the survivors (Dawtry et al., 2020; Pinciotti & Orcutt, 2020). Hence, it seems of utmost importance to encourage caregivers and children to overcome avoidance regarding the CSA incident by providing them with psychoeducation and necessary communication skills.

Affirming the importance of caregiver involvement following CSA, a growing body of literature has established a substantial link between parental invalidation after CSA disclosure and additional deterioration of mental health outcomes in survivors (Hong & Lishner, 2016; Ullman, 2003; Ullman & Peter-Hagene, 2014). More precisely, because children highly depend on their primary caregivers for survival, experiencing parental invalidation or neglect might represent a supplementary existential threat for the affected child (Schmid et al., 2013). Consequently, elevated stress responses due to parental invalidation interfere with thorough memory consolidation post-CSA, therefore further promoting the development of PTSD (Brewin et al., 2010; Elbert & Schauer, 2002; Rukundo-Zeller et al., 2022). Empirical studies found differential effects of trauma-specific versus general parental invalidation on later psychopathology. Hong and Lishner (2016), for instance, found that trauma-specific invalidation in the aftermath of CSA, but not general invalidation, positively predicted subsequent PTSD. These results were recently replicated in a study in the (post-)conflict country Burundi (Schneider et al., 2024). Possibly, maladaptive abuse-related shame coping might cause caregivers to blame the survivor, promoting overgeneralized, exaggerated beliefs about the self, others and the world, thus exacerbating familial conflicts, social exclusion, and trauma symptoms (Côté et al., 2022; Davies & Rogers, 2009; Yasinski et al., 2016). Therefore including caregivers in interventions aiming at trauma recovery seems crucial. The transdiagnostic model (McLaughlin et al., 2020), for instance, emphasizes the protective role of social support, particularly from caregivers, against trauma-related psychopathology. In a Flemish adolescent sample, immediate crisis support post-disclosure was associated with fewer trauma-specific internalizing symptoms compared to no support (Bal et al., 2005). These results concur with previous research evidencing buffering effects of social support following CSA (Bick et al., 2014; Guelzow et al., 2002; Lynskey & Fergusson, 1997; Ullman & Peter-Hagene, 2014). The differential impact of paternal versus maternal validation, though, is controversial. While some studies emphasize the effect of paternal validation after disclosure (Guelzow et al., 2002; Lynskey & Fergusson, 1997), others highlight the importance of maternal validation post-disclosure (Bick et al., 2014; Hébert et al., 2014). Overall, supportive environments and accepting and supportive reactions to CSA disclosures seem essential for recovery. To reinforce emotional support for the child survivors, and to prevent invalidation in the aftermath of CSA by the caregivers, a preventive approach needs to assist the latter to deal with their own emotions. Ultimately, caregivers need to be able to process both their own and their child’s trauma-related reactions, in order to effectively approach and support their child (Yasinski et al., 2016).

Aiming to evaluate the feasibility of a three-session preventive family-communication intervention, including psychoeducation, parental skill training, and PreNET, we collaborated with specialized centers for survivors of sexual violence in Burundi. Having suffered for more than a decade from a civil war and recurring periods of violence and unrest since, Burundi continues to struggle with severe poverty and elevated rates of childhood maltreatment (Charak et al., 2017; Uvin, 2009). Prevailing patriarchal attitudes aggravate gender-based violence (Richter & Dawes, 2008), contributing to substantially elevated rates of CSA (Haro, 2018). The *Centre Seruka*, a specialized center for survivors of sexual violence in the country’s economic capital, Bujumbura, offered services to a total of 3,124 sexually abused Burundian children and adolescents under the age of 13 between the years 2011 to 2015 (Bambonyé & Crombach, 2017). However, sexuality remains a taboo topic, hindering adequate education about sexual abuse. Consequently, caregivers are overwhelmed and distressed when discussing traumatic events (Dyregrov & Yule, 2006), which may lead to many survivors being blamed and rejected for the sexual abuse. Moreover, most survivors are deprived of adequate support, including mental health care, in the aftermath of CSA (Haro, 2018).

These circumstances highlight the need for standardized and effective short-term interventions to prevent the development of PTSD in the aftermath of CSA in (post-)conflict regions. We conducted a study with two cohorts of children and adolescents who had experienced sexual violence, within approximately the past three months. One cohort received treatment as usual in the specialized care centers, the other received our narrative family intervention. We hypothesized that the children of the families who received the intervention would report improved parental acceptance at 3- and 12-month follow-up assessments when compared to the control cohort (Côté et al., 2022; Dawtry et al., 2020; Gopalan et al., 2010; Yasinski et al., 2016). To distinguish between differential effects of the intervention on maternal and paternal acceptance (Bick et al., 2014; Guelzow et al., 2002), we used separate analysis for male and female caregivers. We further hypothesized that we would observe long-term better overall mental health and less severe PTSD symptoms in those children who received the intervention compared to those who did not (Crombach et al., 2015; Elbert & Schauer, 2002; Siehl et al., 2021).

## Method

### Participants

**Fig. 1:**
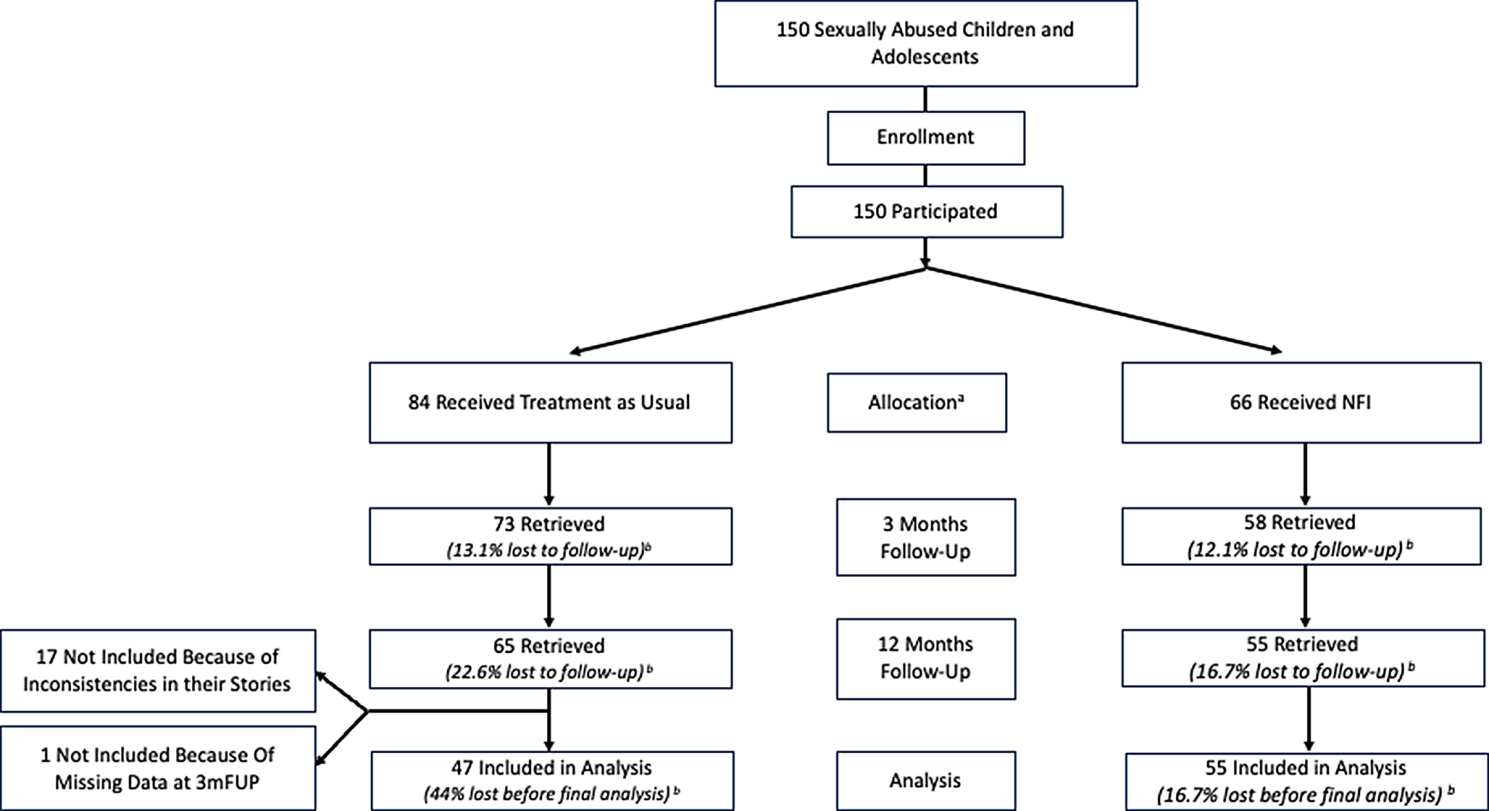
Participant flow-chart with non-randomized allocation. *Note.* NFI = Narrative Family Intervention. ^**a**^ Allocation based on timing of referral. ^**b**^ Attrition rate related to n at allocation

The initial sample included a total of *N* = 150 Burundian children and adolescents with a recent (*M* = 1.7 months, *SD* = 4.0) experience of sexual abuse. While the intervention cohort invariably reported having experienced sexual violence within the last month (*M* = 0.4, *SD* = 0.5, range = 0–1), we observed a greater range within the control cohort (*M* = 3.1, *SD* = 5.5, range = 0–31). In the control cohort, 55.3% had experienced sexual abuse within the last month, while 83% had experienced sexual abuse within the last three months. For the follow-up assessment three months later, we found *N* = 131 (87.3%) of the initial participants, and *N* = 120 (80%) for the interview one year later (see Fig. 1). The final sample consisted of a total of *N* = 102 (*n*_Control_ = 47, *n*_Intervention_ = 55) Burundian female children and adolescents between the ages of 10 and 18 years (*M* = 15.8, *SD* = 1.5). Recruitment took place in collaboration with the first aid center Centre Nturengaho in the provinces Ngozi and Makamba, Burundi. These centers primarily provided care for survivors of sexual violence. The youths included in our study sought support at these centers following an abusive experience. Trained professionals of the centers identified potential participants and invited them to participate after confirming their eligibility. Inclusion criteria required that participants were within the appropriate age range, had experienced sexual abuse within the past months before enrollment, and provided informed consent. Exclusion criteria were severe cognitive impairment - assessed by local psychologists using their clinical judgment - that would have prevented participation in the assessments, or cases who reported incidents which did not align with the study’s definition of sexual abuse. We excluded one person because she participated only in the 12-month, but not the 3-month follow-up. After preparing the data, we found inconsistencies in some participants’ reports of their sexually abusive experience regarding e.g., the number of perpetrators, their relationship to the perpetrator and the setting of the abuse, between initial and follow-up assessments. Furthermore, we found some discrepancies regarding sociodemographic data in these cases.

Ultimately, in these particular cases, we were unable to confirm with certainty that the same individual was interviewed at both follow ups. Hence, as a precaution and to rule out the possibility that we interviewed different survivors at the different time points, we excluded *n* = 17 participants with inconsistent stories from all analyses, knowing we could have also excluded those participants with severe symptom loads.

### Study Design and Procedure

We assessed two cohorts using a longitudinal design. The first cohort (*n* = 84), recruited between March and April 2021, served as a control cohort. The second cohort (*n* = 66), recruited between May and July 2021, received the intervention immediately after the initial assessment (IA). Both cohorts were invited to participate in a 3- and a 12-month follow-up assessment (FUP). Trained local health care workers at Centre Nturengaho and trauma-experienced local psychologists from the Non-Governmental Organization Psychologues sans Frontières Burundi (PSF) carried out the short IA. The psychologists of PSF (co-authors J.-A. M., T. N., L. N, and A. A.) were part of the international team of trauma experts that had developed the intervention. Prior to the IAs and the intervention the psychologists of PSF had trained the local health care workers. The psychologists of PSF had several years of experience in providing trauma-focused psychotherapy. Additionally, they participated in ongoing supervision and consultation with the international trauma experts (co-authors A. C. R.-Z., M. B., and A. C.). Therefore, the intervention and the FUP assessments were conducted by the psychologists of PSF. Both FUP interviews took on average between 1.5 and 2 h with the possibility of breaks, if necessary. To standardize the assessments, all clinicians practiced in joint interviews. Participants with severe PTSD-symptoms in the control cohort (*n* = 2) were offered a NET intervention after the first FUP. However, both participants declined further services.

The present study and all procedures used were approved by the ethics committee of the University of Konstanz and were carried out in accordance with the Declaration of Helsinki. The implementation of this study was supported by the Université Lumière of Bujumbura. Every participant was informed about the study, its objectives, and the procedure. Written informed consent was obtained from all participants and their caregivers prior to the initial screening and again before each FUP interview. They were informed that the data would be used anonymously for scientific purposes and potential publications, and that participation in the study was voluntary. All participants were assured of the confidential handling of personal information, and of no negative consequences regarding access to care independent of the participation in the study. Further, every participant received an individual participant code, so that sensitive information could not be linked to any identifying information. Travel expenses of the participants were paid with research funds (∼5€ per person). The study and its outcome measures were preregistered at Clinical Trials (https://clinicaltrials.gov/) with the registration number NCT05136105.

### Instruments

All instruments used in the semi-structured interviews were translated and back translated between English, French and Kirundi in a double-blinded process using validated versions of the questionnaires. Inconsistencies in the translations were discussed in detail with the entire team, including the translators. The IA was brief because the children had recently experienced CSA, and the IA was designed to be included in the standard assessment of the primary care providers. More detailed questionnaires were reserved for the FUP assessments. All relevant instruments were previously used and validated in studies on mental health problems in Burundi (Crombach et al., 2014; Crombach & Elbert, 2014).

#### Initial Assessment (IA) and Follow-Up Interviews (3-month FUP; 12-month FUP)

##### Sociodemographic Information

 We collected sociodemographic information, including sex, age, birthplace, and educational level, at all three assessments. Further, we asked about the professions of parents or other caregivers. In the event the participants were accompanied by guardians other than their parents, we sought to clarify the nature of their relationship.

##### Sexual Abuse

 We retrieved details about the sexual abuse, including information regarding the date, the location, the exact course of the event, and abuse with and without penetration. Furthermore, we assessed the participant’s relation to, as well as age and sex, of the offender.

##### Abuse-Specific Parental Acceptance

To examine perceived abuse-specific acceptance and rejection by parents or other guardians, all children were asked if they felt accepted by their caregivers immediately after the event, and three and twelve months later. Answer options were *yes* (2), *no* (1), or *no regular contact* (0). For simplicity, we will refer from to female caregivers as mothers and to male caregivers as fathers.

##### Overall Mental Health (SDQ)

Overall mental health was assessed using the Strengths and Difficulties Questionnaire (SDQ; Goodman, 1997) for children aged between 11 and 17. The SDQ comprises a total of 25 items equally divided on five subscales: conduct problems, hyperactivity, emotional symptoms, peer problems, and prosocial behavior. Respondents may indicate their level of agreement on a 3-point Likert-scale ranging from 0 (*not true*) to 2 (*certainly true*). After reverse coding five favorably phrased items, each scale could reach a score between 0 and 10 by summing the item scores. An overall difficulty score with a maximum of 40 can be calculated by adding up all but the prosocial scales (Goodman, 1997; Goodman et al., 2003), with higher scores indicating worse mental health. The questionnaire was previously used (Song et al., 2013) and validated (Zwicknagl, 2020) in Burundi. For the Kirundi version of the SDQ in a female sample, cut-off scores ranged from 14 to 16 for the 80th to 90th percentiles and above 16 for the 90th to 100th percentiles. Zwicknagl (2020) reported a Cronbach’s α of 0.67 in a sample of 397 Burundian adolescents between the ages of 11 to 17 years. Our sample yielded Cronbach’s α of 0.62 at the IA.

#### Follow-Up Interviews Only (3-month FUP; 12-month FUP)

PTSD Symptom Severity. PTSD symptoms were examined using the University of California Los Angeles (UCLA)-PTSD Reaction Index (RI; Steinberg et al., 2004; Steinberg et al., 2013) for children and adolescents based on the DSM-5 criteria (Pynoos & Steinberg, 2015). The questionnaire includes 31 items which are scored on a 5-point Likert scale ranging from 0 (*none of the time*) to 4 (*all of the time*) for the past month. An overall PTSD severity score ranging from 0 to 80 can be calculated by summing the individual symptom scores excluding dissociative features according to scoring protocols (Pynoos & Steinberg, 2013). Higher scores indicate more PTSD-symptoms. The DSM-5 RI has been successfully utilized and validated in non-western low-income countries (Doric et al., 2019; Kaplow et al., 2020). The DSM-5 RI has yielded a Cronbach’s α of 0.93 in a sample of 33 Burundian male children and adolescents between 12 and 20 years of age (Rukundo-Zeller et al., 2022). Cronbach’s alpha of the symptom checklist in our sample was excellent with α = 0.91 at the 3-month FUP.

### Intervention

**Fig. 2:**
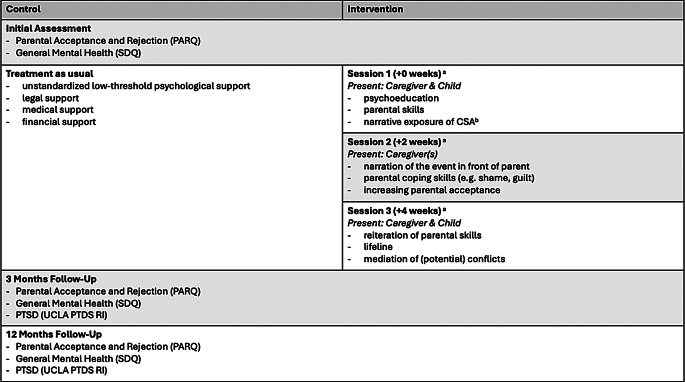
Narrative family intervention. Note. CSA = Childhood Sexual Abuse. ^**a**^ Time data in relation to initial assessment. ^**b**^ The NET intervention was conducted with the child only. Parents were asked to leave the room. Exceptions were made if the psychologists thought emotional support during the exposure was necessary

The intervention had three objectives: (1) preventing the build-up of the fear-network, (2) preventing avoidant behaviors of both the affected child and the family, and (3) preventing parental rejection. The intervention consisted of three sessions of about 2–3 h, including breaks if necessary. The first session took place directly after the IA with both, the (non-offending) caregiver and child, during their first visit to the specialized care center. Only the caregivers were invited for a second session two weeks later. Another two weeks later, the caregivers and the child were invited for the third and final session (see Fig. 2).

During the first session, the caregiver (s) and the child were informed about the objective and the duration of the intervention. They received psychoeducation about the importance of parental validation and how caregivers could support the child when the child mentioned or disclosed the sexual abuse to them: (1) Caregiver(s) were encouraged and instructed how to signal interest and compassion; (2) the importance of properly addressing their own emotions, such as reacting authentically but not overwhelming the child with their own struggles and remaining in charge of the situation; (3) the importance of being ready to signal support by saying, for example, that they love/like/value their children or offering hugs if the child wanted them. Moreover, they received information about traumatic memories, the fear network, and why it might be beneficial for the child to narrate the recent sexual incident in detail. Subsequently, the child was encouraged to narrate the recent incident of sexual violence to the psychologist, following the logic of NET. This part of the session was conducted with the child alone, except if the caregiver’s emotional support was necessary in the opinion of the psychologist. After the narrative exposure, the child was encouraged to remind themselves every time they experienced an intrusion when and where the incident happened. Furthermore, the child was asked to provide consent that the narration could be read during the second session to the caregivers. In the end of the first session, the caregiver(s) received further psychoeducation about common emotional and behavioral reactions in child survivors of sexual abuse, and how caregivers could support and encourage the child to overcome avoidance, guilt, and isolation. This psychoeducation was supported by a detailed flyer. Furthermore, measures taken by the caregiver(s) to ensure the future safety of the child were discussed. For the next appointment explicitly both principal caregivers were invited.

The second session was conducted ideally with both caregivers. The principal objective of this session was to assist the caregivers in processing their own emotions regarding the sexual abuse of their child. Furthermore, this session sought to reduce (voluntary and involuntary) rejection or invalidation of the child by the caregivers resulting from the recently experienced sexual abuse. In a first step, the narration of the child was read to the caregivers. If the child had not given their consent, the caregivers were asked instead to share what they knew about the sexual abuse. Then the caregivers were encouraged to express how they felt when hearing this detailed narration of their child and the traumatic incident. In a second step, the psychologist provided psychoeducation regarding the emotions that arose, including normalizing them, understanding the reasons for them, and how they might impact the child and the family. Then they provided guidance in ways of effectively processing them. Most importantly, the session focused on helping the caregivers understand and address shame. Shame is a highly aversive feeling associated with social rejection, which is rarely confronted (Gilbert, 2000; Lewis, 2003). Referring to the compass of shame (Elison et al., 2006) frequently used strategies to avoid experiencing shame were explained. In particular, the risk of blaming the survivor, i.e., the child, to avoid a parent’s own feelings of humiliation, shame, and helplessness, was emphasized (Dawtry et al., 2020; Stuewig et al., 2010). Through compassionately understanding and reflecting the shame of the caregivers, the psychologists aimed to assist the caregivers in overcoming maladaptive shame coping strategies, and to reinforce the understanding that blaming the child instead of the aggressor might not be a helpful reaction. Moreover, reactions of the child during the sexual abuse were discussed and explained, including dissociative reactions, and why the child might not have defended herself (Schauer & Elbert, 2015). Finally, the psychologist helped the caregivers to deal with feelings of guilt using cognitive techniques. At the end of the session, the caregivers were invited to share again how they felt. Furthermore, they were reminded of the strategies learned in the first session to support their child.

In the third session both, the caregiver(s) and the child were present. In line with the logic of NET, the child was invited to put down their lifeline. The caregivers could assist the child with early events, and in terms of chronology. The aim of this exercise was to place the recent sexual abuse chronologically within the biography of the child, and to improve the understanding of reactions during and after the new traumatic experience. Once the child reached the sexual abuse, the lifeline was halted, and the psychologist read the narration of this abuse again to the child and the caregiver(s). The caregiver(s) were encouraged and assisted in showing supportive behavior during this re-reading of their child’s narrative using skills learned in the previous sessions. Then the child finished the lifeline. Afterwards, the child was invited to ask their caregiver(s) questions regarding the sexual abuse. Likewise, the caregiver(s) were encouraged to answer the questions, and to ask questions themselves. If needed, the psychologists provided further explanations and psychoeducation. In case the lifeline revealed other events that needed explanations, such as severe punishments by the caregivers, the child and the caregiver(s) were given the opportunity to discuss them to avoid potential conflicts after the end of the intervention. To conclude the intervention the psychologist reiterated the skills the family had learned. They emphasized safety provisions taken by the family, and the right of the child to say “no” to any unwanted body contact. Moreover, they encouraged the family to continue supporting each other.

The control cohort participated only in the standardized assessments. In addition, they received treatment as usual at the specialized care center. That included unstandardized low-threshold psychological support, a single listening session, alongside legal, medical, and financial support (see Fig. 2). Nevertheless, the limited infrastructure and logistical challenges in rural areas of Burundi resulted in most participants not receiving any treatment.

### Data Analysis

To examine whether there were statistically significant changes regarding parental acceptance at all three assessment times between and within both cohorts, we performed an aligned rank transform analysis of variance (ART ANOVA; Elkin et al., 2021; Higgins et al., 1990) for both mothers and fathers separately using the statistics software R version 2021.09.2 with the package “ARTool”. For these analyses, we excluded additional *n* = 6 participants from the analyses for maternal, and *n* = 4 for paternal acceptance, because they changed the person they identified as primary caregiver throughout the project. The remaining data analyses were carried out using the statistical program SPSS version 28. We calculated a mixed model ANOVA to evaluate cohort differences regarding overall mental health and PTSD symptoms. Because of violations of assumptions, a robust mixed ANOVA was performed for the evaluation of PTSD. We found no differences in statistical significance between the robust- and non-robust ANOVA, hence we reported the results from the non-robust analysis. Violations of the normal distribution were accepted due to the robustness of the analysis (Harwell et al., 1992; Lumley et al., 2002). In the case of outliers, all analyses were performed again without outliers. Since no differences in statistical significances emerged, outliers were kept in the data, assuming they were true values. In case of non-significant interaction effects, we still calculated simple main effects to obtain effect sizes for longitudinal within-cohort effects regarding overall mental health. To account for multiple testing, *p*-values for post-hoc analyses were adjusted using the Bonferroni-Holm method. Due to directional hypothesis regarding the effect of our intervention, a one-tailed significance level of α < 0.05 was assumed for all interaction analyses and analyses of follow-up effects of the intervention cohort. Effect sizes for pairwise comparisons were estimated using Cohen’s *d* (Cohen, 1988). Effect sizes for repeated measures (*d*_rm_) were adapted according to Morris (2008). For effect sizes, 95% confidence intervals were computed. We calculated reliable change indices (Jacobson & Truax, 1991) to evaluate significant changes in scores on an individual level. Reliable change indices higher than 1.65 were counted as significant because of directional hypotheses. Aiming to verify the robustness of the results, we conducted all analyses again using different inclusion criteria for our analyses: (1) In addition to the per-protocol-analyses, we conducted intention-to-treat-analyses, which usually yield more conservative results (Abraha et al., 2015; McCoy, 2017). However, due to the particularities of this study, we considered the per-protocol-analyses more adequate. Firstly, all participants had completed the intervention. Secondly, dropout in our study was primarily driven by logistical and geographical challenges rather than systematic differences related to symptom severity or condition assignment. Thirdly, we lost more participants of the control cohort than the intervention cohort. (2) We conducted additional analyses including the full sample, i.e., including the 17 participants we had excluded due to inconsistencies in their stories, as we might have excluded the most severely affected participants. (3) We conducted additional analyses excluding all individuals who had experienced the sexual abuse more than a month prior to the IA (remaining sample: intervention cohort *n* = 55; control cohort *n* = 26). Thereby, we took the literature into consideration that suggests PTSD symptoms tend to stabilize 3 months after sexual assaults (Oosterbaan et al., 2019).

## Results

### Descriptive Statistics

At IA, participants completed on average 4.9 years of education (*SD* = 2.8, range = 0–10). Nearly a quarter of the participants reported still attending school, whereas 73.5% either never went or stopped attending school. One survivor did not give any answer regarding their current educational status. Of the survivors, 95.1% reported having experienced a sexual abuse with penetration, and 66.7% of all survivors reported knowing their perpetrator. See Table 1 for detailed sociodemographic data split up per cohort.

**Table 1:** Descriptive statistics of participants per Study-Cohort

	Control			Intervention		
	*M*	*SD*	Range	*M*	*SD*	Range
Age	16.4	0.9	14–18	15.2	1.7	10–17
Educational Level	5.3	3.0	0–10	4.5	2.5	0–10
Age of Offender	27.2	6.4	18–40	30.6	8.6	18–60
Time since Abuse (Months)	3.1	5.5	0–31	0.4	0.5	0–1
Life Events	5.4	2.4	1–11	4.5	2.3	1–11
SDQ-Overall (IA)	13.7	5.3	6–27	15.4	4.4	4–26
SDQ-Overall (3mFUP)	11.7	4.6	4–24	10.3	4.9	1–26
SDQ-Overall (12mFUP)	13.6	4.9	4–24	11.5	4.5	3–19
UCLA-PTSD-Overall (3mFUP)	16.7	12.9	0–52	7.8	7.0	0–38
UCLA-PTSD-Overall(12mFUP)	11.1	11.4	0–62	4.0	3.3	0–13

*Note. N*_*Control*_ = 47, *N*_*Intervention*_ = 55. SDQ = Strengths and Difficulties Questionnaire summed up (0–40), UCLA-PTSD = University of California - Posttraumatic Stress Disorder - Reaction Index summed up (0–80). IA = Initial Assessment, 3mFUP = 3-month follow-up, 12mFUP = 12-month follow-up

### Parental Validation

**Fig. 3:**
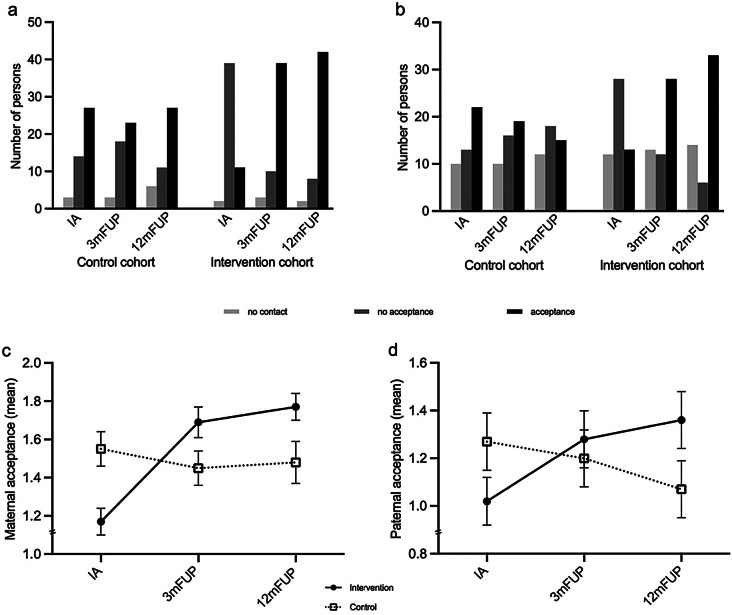
Frequencies of specific parental acceptance. Note. **a**) and **c**): for maternal acceptance: N_Control_ = 44, N_Intervention_ = 52; b) and d) for paternal acceptance: N_Control_ = 45, N_Intervention_ = 53. **a**) and **b**) illustrate the counts of the reported acceptance categories, **c**) and **d**) illustrate the means of the reported acceptance categories, with higher means indicating more acceptance. IA = Initial Assessment, 3mFUP = 3-month follow-up, 12mFUP = 12-month follow-up. Error bars represent standard errors

Regarding maternal validation the ART ANOVA showed a significant main effect for cohort (*F* (1, 94) = 8.41, *p* =.005, η_p_² = 0.08, 95% CI [0.01, 0.20]), time (*F* (2, 188) = 11.47, *p* <.001, η_p_² = 0.11, 95% CI [0.03, 0.19]), and a significant time*cohort interaction effect (*F* (2, 188) = 6.68, *p* =.002, η_p_² = 0.07, 95% CI [0.01, 0.14]), indicating a stronger improvement of maternal acceptance in the intervention cohort (see Fig. 3a and c). Post-hoc contrast tests with Bonferroni-Holm-corrected *p*-values revealed a statistically significant difference in maternal acceptance at IA (*t*(282) = 52.31, *p* =.003) but not at 3-month FUP (*t*(282) = -31.19, *p* =.103) nor at 12-month FUP (*t*(282) = -31.93, *p* =.101). We found statistically significant improvements in perceived maternal acceptance over time in the intervention cohort between both IA and 3-month FUP (*t*(188) = 71.28, *p* <.001) and between IA and 12-month FUP (*t*(188) = 80.18, *p* <.001) with Bonferroni-Holm-corrected *p*-values. No statistically significant differences regarding perceived maternal acceptance were found for the control cohort over time, neither between IA and 3-month FUP (*t*(188)= -12.23, *p* >.999) nor between IA and 12-month FUP (*t*(188) = -4.06, *p* >.999).

Regarding paternal validation the ART ANOVA showed no significant main effect for cohort (*F* (1, 96) = 0.01, *p* =.932, η_p_² = 0.00, 95% CI [0.00, 0.00]), but a significant main effect for time (*F* (2, 192) = 6.51, *p* =.002, η_p_² = 0.06, 95% CI [0.01, 0.13]), and a significant time*cohort interaction effect (*F* (2, 192) = 4.82, *p* =.009, η_p_² = 0.05, 95% CI [0.00, 0.11]), indicating a stronger improvement of paternal acceptance in the intervention cohort (see Fig. 3b and d). Post-hoc contrast tests with Bonferroni-Holm-corrected *p*-values revealed no statistically significant difference between cohorts in paternal acceptance at IA (*t*(187) = 27.51, *p* =.995) nor at 3-month FUP (*t*(187) = -9.94, *p* =.500) or 12-month FUP (*t*(187) = -32.46, *p* =.281). However, we found statistically significant improvements in perceived paternal acceptance over time in the intervention cohort between both IA and 3-month FUP (*t*(192) = 30.01, *p* =.036) and between IA and 12-month FUP (*t*(192) = 38.98, *p* =.003) with Bonferroni-Holm-corrected *p*-values. No statistically significant differences were found for the control cohort over time, neither between IA and 3-month FUP (*t*(192)= -7.43, *p* >.999) nor between IA and 12-month FUP (*t*(192) = -20.99, *p* =.833). Cohort by time interaction effects regarding maternal and paternal acceptance remained statistically significant in the intention-to-treat-analysis, the full sample, and the smaller sample excluding all individuals who had experienced the sexual abuse more than a month prior to the IA.

### Overall Mental Health

**Fig. 4:**
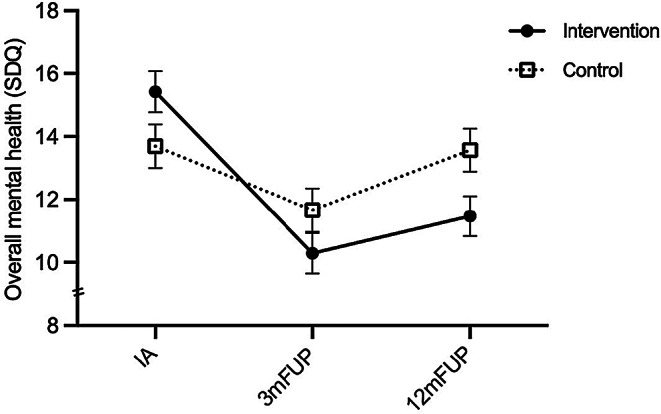
Comparison of overall mental health per cohort. Note. Mixed model analysis comparing the mental health changes per cohort between initial assessment, 3-month follow-up, and 12-month follow-up using the SDQ overall scores. SDQ = Strengths and Difficulties Questionnaire summed up (0–40). N_Control_ = 47, N_Intervention_ = 55. IA = Initial Assessment, 3mFUP = 3-month follow-up, 12mFUP = 12-month follow-up. Error bars represent standard errors

Regarding overall mental health the mixed model ANOVA showed no significant main effect for cohort (*F*(1, 100) = 0.95, *p* =.332, η_p_² = 0.01, 95% CI [0.00, 0.08]), but a significant main effect for time (*F*(2, 200) = 16.22, *p* <.001, η_p_² = 0.14, 95% CI [0.06, 0.22]), and a significant time*cohort interaction effect (*F*(2, 200) = 5.15, *p* =.007, η_p_² = 0.05, 95% CI [0.00, 0.11], see Fig. 4). Bonferroni-Holm corrected post-hoc between cohort comparisons over time revealed no statistically significant differences between the cohorts at IA (*p* =.078, *d* = 0.36, 95% CI [-0.04, 0.75]), at 3-month FUP (*p* =.151, *d* = 0.29, 95% CI [-0.68, 0.10]) and at 12-month FUP (*p* =.151, *d* = 0.45, 95% CI [-0.84, -0.05]). Bonferroni-Holm corrected post-hoc within cohort comparisons over time revealed an improvement of overall mental health symptoms within the intervention cohort between IA and 3-month FUP (*p* <.001; *d*_rm_ = -0.81, 95% CI [-0.92, -0.14]) and between IA and 12-month FUP (*p* <.001; *d*_rm_ = -0.66, 95% CI [-0.87, -0.10]). No significant improvements were found for the control cohort between IA and 3-month FUP (*p* =.092 *d*_rm_ = -0.33, 95% CI [-0.70, 0.11]) and between IA and 12-month FUP (*p* =.903 *d*_rm_ = -0.02, 95% CI [-0.42, 0.39]). The results regarding overall mental health remained statistically significant in all analyses using the intention-to-treat analysis (intervention cohort: IA und 3mFUP *d*_rm_ = -0.74 and IA und 12mFUP *d*_rm_ = -0.60; control cohort: IA und 3mFUP *d*_rm_ = -034 and IA und 12mFUP *d*_rm_ = -0.05), the full sample, and the smaller sample excluding all individuals who had experienced the sexual abuse more than a month prior to the IA.

According to the reliable change index, results on the individual level showed a similar direction regarding overall mental health. Between the IA and the 3-months FUP, 12 (21.8%) participants of the intervention cohort reported an improvement, and 1 (1.8%) participant reported a deterioration of overall mental health. Between the IA and the 12-months FUP, 8 (14.5%) participants reported an improvement, and 0 participants reported a deterioration of overall mental health. In contrast, 3 (4.3%) participants of the control cohort reported an improvement, and 1 (2.1%) participant reported a deterioration of overall mental health symptoms between the IA and the 3-month FUP. Between the IA and the 12-month FUP, 2 (4.3%) participants in the control cohort reported an improvement, and 2 (4.3%) participants reported a deterioration of overall mental health symptoms.

### PTSD Symptoms

Another mixed model ANOVA examining the change of posttraumatic stress symptoms over time between the 3-month and the 12-month FUP, found significant main effects of cohort, *F*(1, 100) = 28.71, *p* <.001, η_p_² = 0.22, 95% CI [0.09, 0.35], and of time, *F*(1, 100) = 19.69, *p* <.001, η_p_² = 0.17, 95% CI [0.05, 0.29]. There was no significant time*cohort interaction effect, *F*(1, 100) = 0.72, *p* =.200, η_p_² = 0.01, 95% CI [0.00, 0.07]. Cohorts differed significantly at 3-month FUP, *t*(68.39) = 4.20, *p* <.001, *d* = 0.87, 95% CI [0.46, 1.28], and at 12-month FUP, *t*(52.70) = 4.12, *p* <.001, *d* = 0.87, 95% CI [0.46, 1.28]. The intervention cohort reported significantly lower PTSD symptoms at 3-month (*M*_*3mFUP*_ = 7.8, *SD*_*3mFUP*_ = 7.0) and 12-month FUPs (*M*_*12mFUP*_ = 4.0, *SD*_*12mFUP*_ = 3.3), than the control cohort (*M*_*3mFUP*_ = 16.7, *SD*_*3mFUP*_ = 12.9; *M*_*12mFUP*_ = 11.1, *SD*_*12mFUP*_ = 11.4). In both cohorts, PTSD symptoms improved from the 3-month to the 12-month FUP (intervention cohort: *d*_*rm*_ = -0.45, 95% CI [-0.83, -0.07]; control cohort: *d*_*rm*_ = -0.38, 95% CI [-0.77, 0.05]). The results regarding PTSD symptom severity were similar using the intention-to-treat analysis (intervention cohort: 3mFUP und 12mFUP *d*_rm_ = -0.46; control cohort: 3mFUP und 12mFUP *d*_rm_ = -0.29), and the full sample. Main effects of cohort but not of time on PTSD symptom severity remained statistically significant in the analysis using the smaller sample excluding all individuals who had experienced the sexual abuse more than a month prior to the IA.

## Discussion

We demonstrated the feasibility of implementing a narrative family intervention to prevent a deterioration of mental health symptoms in child survivors of CSA in Burundi, a low-income country which has suffered from severe violence in the past. In a cohort design, we found that maternal and paternal acceptance improved significantly over time in the intervention cohort, while no changes were observed within the control cohort. Similarly, overall mental health symptoms improved over time in the intervention cohort, but not in the control cohort. PTSD symptom severity was significantly lower at both follow-up assessments in the intervention cohort compared to the control cohort. The courses of overall mental health symptoms as well as PTSD symptoms in the intervention cohort suggest that the improvements remained sustainable over the period of one year. All results proved to be robust using different analyzing strategies and inclusion criteria, such as excluding all participants of the control cohort who reported that the sexual abuse happened more than a month prior to the initial assessment.

We suggest that we were able to promote parental acceptance by explaining and normalizing psychological and behavioral consequences of CSA in both, the children and the parents (Borbé et al., 2017; Liedl et al., 2013). By learning how to process their own trauma-related emotions and learning coping strategies, parents were less likely to blame their child for the abuse (Stuewig et al., 2010). Rather, parents were able to engage in a supportive relationship with their offspring. Additionally, the increasing level of acceptance in the intervention cohort indicates that we were able to reinforce the parents’ knowledge that a validating relationship can substantially and positively affect the symptom course following CSA (Lynskey & Fergusson, 1997; Ullman & Peter-Hagene, 2014). Possibly, the improvement of paternal acceptance is an indicator that families exchanged about the sexual abuse due to our intervention, as fathers were less likely to show up for the parent session of the intervention than mothers. This possible pathway is supported by the fact that, in the control cohort, paternal acceptance decreased throughout the project. This decrease in acceptance might be a manifestation of the fathers’ own distress and inability to cope with the CSA of their child. Consequently, fathers might have started to avoid or blame their offspring (Dawtry et al., 2020; Yasinski et al., 2016), which might have given the children in the control cohort the impression they were not being accepted.

Furthermore, we observed a significant improvement in overall mental health difficulties over time in those individuals who received the intervention. We suggest that enhanced intrafamilial communication in the intervention cohort may have prompted reappraisal of the incident not only on the individual but on the familial level (Dunmore et al., 2001; Ehlers & Clark, 2000). Hence, the intervention might have effectively counteracted negative cognitions and avoidance within the family. By employing psychoeducation and parental communication training we might have further averted maladaptive coping mechanisms such as parental shame or victim-blaming, thereby mitigating potential family conflicts and social isolation of the survivor (Côté et al., 2022; Davies & Rogers, 2009). By assisting parents in the processing of their own trauma-related reactions alongside their child’s trauma-related reactions, we hypothesize that we have supported the creation of space within the family environment for the survivor to process and cope with the abuse (Yasinski et al., 2016), resulting in an improvement of mental health within the first year after the abuse. Our results align with existing literature showing that parental support post-CSA is linked to fewer mental health symptoms (Bick et al., 2014; McLaughlin et al., 2020; Yasinski et al., 2016).

Although we did not obtain initial PTSD symptom severity, our results provide additional tentative evidence that the intervention cohort was significantly less affected during the follow-up assessments than was the control cohort. In line with the theoretical background of the fear network, the narrative family intervention (NFI) procedure might have successfully reconnected event-related emotions with their context through detailed narrations of the abusive event (Brewin et al., 2010; Elbert & Schauer, 2002). The improvement of overall mental health supports this assumption, as previous findings showed significant associations between posttraumatic stress and emotional as well as conduct problems (Barboza et al., 2017; Grassetti et al., 2018; Haller & Chassin, 2012; Logue et al., 2013; Miller & Resick, 2007). Typically, PTSD symptoms tend to stabilize around 3 months after a traumatic event, underscoring the importance of timely intervention (Oosterbaan et al., 2019). For instance, according to the literature, within the first week after a sexual assault up to 94% of survivors meet diagnostic criteria for acute stress disorder, which has similar diagnostic criteria as PTSD excepting the duration of the symptoms. This percentage decreases to 45–80% within the first three months (Elklit & Christiansen, 2010; Rothbaum et al., 2006; Steenkamp et al., 2012). It may be that the high number of experienced traumatic events may have interfered with the spontaneous remission of the control cohort (Kolassa et al., 2010). However, due to our non-random assignment of participants, and not having assessed the initial PTSD symptom load prior to our intervention, we cannot definitively attribute the results to the efficacy of our family-oriented approach.

The following caveats limit the interpretation of the results. Our cohort design lacked random assignment to either the control or intervention cohort. Hence, we cannot exclude the possibility that sampling effects may have impacted our findings. Despite this possibility, there was no indication suggesting that the time points of recruitment could have an impact on the outcomes of this study as the two cohorts differed only regarding maternal acceptance at baseline assessment, which we controlled for statistically. However, since the control group received treatment as usual (i.e., unstandardized, low-threshold support), the lack of standardized treatments may have contributed to the observed group differences, limiting the ability to attribute the effects solely to the intervention. Possibly, placebo effects associated with common factors of psychotherapeutic interventions such as empathy and hope might have contributed to some of the improvements of the intervention group (Cuijpers, 2012). To provide more stringent evidence for the effect of the intervention under controlled conditions, a future randomized controlled trial (RCT) would be necessary. Furthermore, due to the logistics of organizing the follow-up assessments, the interviewers were not blinded regarding the cohort assignments. Further, the control cohort reported a wide range of months elapsed since the abuse, while the intervention cohort reported having experienced the abuse at maximum one month prior to the initial assessment. Nevertheless, we assume that our results are robust, as we evaluated this divergence by conducting the statistical analyses again with a subsample of those who reported having experienced the abuse one month prior to the initial assessment. Data of this study was collected in collaboration with two rural agencies of a primary care center for survivors of sexual violence. More diverse environmental settings and ideally a multi-center study might facilitate conclusions regarding the generalizability of this approach, and its potential for dissemination. On the other hand, we would like to point out, that we tested the feasibility of the intervention in challenging conditions outside of urban settings with very little infrastructure, which could be interpreted as an indicator for the feasibility of the approach. Lastly, our sample comprised exclusively females, limiting the generalizability of our interpretations to the female gender.

While late interventions following CSA are thoroughly researched and their effectiveness is well documented (Regehr et al., 2013; Vickerman & Margolin, 2009), data on early interventions is still limited, hindering definite conclusions about the efficacy thereof (Oosterbaan et al., 2019). With our research, we could demonstrate the feasibility and potential effectiveness of an early intervention to prevent mental health deterioration in the aftermath of CSA. Furthermore, we contributed to the development of a standardized preventive intervention program in the aftermath of CSA in resource-poor regions.

## Data Availability

Due to the highly sensitive nature of this research, we did not make their data available in a data repository. However, participants gave their informed consent that excerpts of the data that guarantee anonymity can be shared with other researchers upon reasonable request. Please address requests to the corresponding author.
